# Strain-Specific Benefits of *Bacillus* Probiotics in Hybrid Grouper: Growth Enhancement, Metabolic Health, Immune Modulation, and *Vibrio harveyi* Resistance

**DOI:** 10.3390/ani14071062

**Published:** 2024-03-30

**Authors:** Congjie Han, Shizhen Song, Congcong Cui, Yan Cai, Yongcan Zhou, Jiawen Wang, Weilie Bei, Dongdong Zhang, Weiliang Guo, Shifeng Wang

**Affiliations:** 1 Hainan Provincial Key Laboratory for Tropical Hydrobiology and Biotechnology, School of Marine Biology and Fisheries, Hainan University, Haikou 570228, China; 2 Collaborative Innovation Center of Marine Science and Technology, Hainan University, Haikou 570228, China

**Keywords:** *Bacillus subtilis*, grouper, growth, immunity, lipid metabolism, disease resistance

## Abstract

**Simple Summary:**

Probiotics, beneficial bacteria that contribute to the health of both humans and animals, play a crucial role in aquaculture by enhancing digestion and bolstering disease resistance in fish. However, the efficacy of probiotics can vary significantly, even among strains of the same species, based on their origin. This study focused on evaluating the effects of three different *Bacillus subtilis* strains, derived either from the host fish or external sources, on fish growth and disease resilience. Our findings indicate that the host-derived strain, 6-3-1, offered superior benefits in terms of growth enhancement and health improvement compared to the externally-derived strains. This research underscores the importance of the probiotic source, demonstrating that origin can significantly influence effectiveness. The outcomes suggest the potential for customized probiotic applications to advance aquaculture practices substantially.

**Abstract:**

In the realm of modern aquaculture, the utilization of probiotics has gained prominence, primarily due to their ability to enhance growth, boost immunity, and prevent diseases in aquatic species. This study primarily investigates the efficacy of *Bacillus subtilis* strains, both host-derived and from other sources, in influencing fish growth, immunity, lipid metabolism, and disease resistance. Employing a 42-day feeding trial, we divided hybrid grouper into four distinct groups: a control group on a basal diet and three experimental groups supplemented with 1 × 10^8^ CFU/g of different *Bacillus subtilis* strains-BS, 6-3-1, and HAINUP40. Remarkably, the study demonstrated that the 6-3-1 and HAINUP40 groups exhibited significant enhancements across key growth parameters: final body weight (FBW), weight gain rate (WGR), feed intake (FI), feed efficiency ratio (FER), and feed conversion ratio (FCR). The investigation into lipid metabolism revealed that the 6-3-1 strain upregulated seven metabolism-related genes, HAINUP40 affected four metabolism-related genes, and the BS strain influenced two metabolism-related genes, indicating diverse metabolic impacts by different strains. Further, a notable reduction in liver enzymes AST and ALT was observed across all supplemented groups, implying improved liver health. Noteworthy was the BS strain’s superior antioxidative capabilities, positively affecting all four measured parameters (CAT, GSH-Px, MDA). In the sphere of immune-related gene expression, the BS strain significantly decreased the expression of both inflammation and apoptosis-related genes, whereas the HAINUP40 strain demonstrated an upregulation in these genes. The challenge test results were particularly telling, showcasing improved survival rates against *Vibrio harveyi* infection in the BS and 6-3-1 groups, unlike the HAINUP40 group. These outcomes highlight the strain-specific nature of probiotics and their varying mechanisms of action within the host. In conclusion, this study reveals that probiotic strains, varying by source, demonstrate unique, strain-specific effects in promoting growth and modulating immunity in hybrid grouper. This research highlights the promise of tailored probiotic applications in improving aquaculture practices. Such advancements contribute to more sustainable and efficient fish farming methods.

## 1. Introduction

Aquaculture, currently the world’s fastest-growing animal production sector, trades approximately 160 million tons of farmed fish annually, valued at around USD 80 billion [1,2,3]. This upward trend is particularly notable in high-value demersal fish like groupers [4,5]. Projections indicate a 30% increase in aquaculture demand in Asia and the Pacific by 2030. To meet this demand sustainably, Asian countries must adopt ecofriendly practices that adhere to industry standards, fostering responsible sector growth while protecting the environment [6]. The hybrid grouper (*Epinephelus fuscoguttatus*♀ × *Epinephelus lanceolatus*♂) has gained popularity in Southeast Asian aquaculture. Its resilience to high-density farming and rapid growth makes it an ideal candidate for intensive cultivation [7,8,9]. However, hyperintensive farming practices have led to reduced growth efficiency and an increase in disease infections in these fish, posing significant challenges to the industry [10]. Traditional strategies to mitigate intensified farming issues, such as using antimicrobials and chemicals to prevent disease, have drawbacks [11]. These agents alter the intestinal and aquatic microbial communities, hinder host immune development, and potentially impact human health due to antibiotic residues and bacterial resistance [11,12,13]. Given these challenges, it is crucial to explore alternative disease control methods that eschew therapeutic chemicals and antibiotics. Such methods should enhance survival, promote growth, and bolster disease resistance in aquaculture [14].

Probiotics, recognized as effective alternatives for disease prevention and control in aquaculture [15], comprise live microorganisms capable of adapting to and proliferating in the host’s intestinal tract. Recent years have highlighted their benefits in aquaculture, notably in enhancing growth performance, disease control, immune response improvement, and digestive enzyme activity [16,17]. Among these, *Bacillus* species are widely employed as probiotics in aquatic animals [18,19,20,21]. Studies have demonstrated that probiotics do not lead to drug resistance in aquatic species, making them viable antibiotic substitutes [22,23,24]. 

Presently, a diverse array of probiotic strains is utilized. However, research indicates that the efficacy of these strains is highly specific to each strain. Notable differences in coaggregation, cell adhesion, growth inhibition, and duodenal gene expression have been observed among strains of the same bacterial species, even under identical supplementation protocols and conditions [25,26,27,28]. Furthermore, given the specificity of host microbial communities, it is hypothesized that probiotics derived from the host may be more effective in the host environment than those from other sources [29]. Yet, comparative studies on the in vivo effects of host-derived and externally derived strains are scarce.

In a previous study, we identified three potential *Bacillus subtilis* probiotic strains (BS, 6-3-1, and HAINUP40) from varied sources. The BS strain, isolated from shrimp paste, exhibited promising probiotic characteristics in vitro [30]. The 6-3-1 strain, derived from hybrid grouper intestines, effectively enhanced growth and boosted both nonspecific immune responses and disease resistance in hybrid grouper [31]. The HAINUP40 strain, sourced from natural pond water, improved growth, digestive enzyme activity, nonspecific immunity, and disease resistance in Nile tilapia (*Oreochromis niloticus*) [32]. This study aims to compare the modulatory mechanisms of different *B. subtilis* strains on host fish genes and assess whether host-derived strains are more effective than those from other sources. By examining the impacts on fish growth, immunity, lipid metabolism, and disease resistance, this research yields insights valuable for the application of probiotics in the healthy and sustainable farming of grouper fish.

## 2. Experimental Design and Methods

### 2.1. Experimental Location and Holding Preparation

The study was conducted at a cooperative grouper farming base in Haitou Town, Danzhou City, Hainan Province, China. We acclimated 360 healthy hybrid groupers in four 1000 L tanks over 14 days. During this period, the fish were given balanced feed (the same as basal feed) to meet their nutritional needs and facilitate environmental adaptation. The aquatic environmental parameters under acclimation conditions were the same as those in the feeding experiment.

### 2.2. Fish Growth Parameters Measurement

At the experiment’s start and end, we measured the initial body weight (IBW) and final body weight (FBW) of ten randomly selected fish from each group. We also calculated feed intake (FI, g/day), feed efficiency rate (FER, %), feed conversion rate (FCR, %), and weight gain rate (WGR, %), using the following formulas: FI = feed intake/duration (days); FER = 100 × (FBW − IBW)/feed intake; FCR = 100 × feed intake/(FBW − IBW); WGR = 100 × (FBW − IBW)/IBW.

### 2.3. Preparation of Probiotic Diet

In our laboratory, we employed three *Bacillus subtilis* strains-BS, 6-3-1, and HAINUP40-as probiotics. These strains were isolated and identified by our team: BS was derived from traditional Chinese fermented shrimp paste [30], 6-3-1 from the intestines of hybrid grouper (*Epinephelus fuscoguttatus*♀ × *Epinephelus lanceolatus*♂) [31], and HAINUP40 from natural pond water [32]. For the preparation of probiotic-supplemented diets, each *Bacillus* strain was cultured in liquid LB medium and incubated at 37 °C for 24 h. Following incubation, the cultures were centrifuged, and the resulting pellets were resuspended in sterile PBS. We then sprayed these probiotic suspensions evenly onto basal feed pellets to achieve a concentration of 1 × 10^8^ CFU (colony-forming units) bacteria per gram of feed. For the control group, basal feed was sprayed with a comparable volume of sterile PBS. The final experimental diets included: a BS diet (basal feed with BS strain), a 6-3-1 diet (basal feed with 6-3-1 strain), a HAINUP40 diet (basal feed with HAINUP40 strain), and a control diet (basal feed with PBS only). After spraying, the diets were dried in a bacterial oven at 30 °C for 12 h. This process was conducted on a weekly basis. Post-preparation, the diets were portioned into plastic bags and stored at −20 °C until needed. The basic diet’s ingredients and approximate composition are listed in Table 1.

### 2.4. Fish Rearing and Experimental Design

In this study, we distributed 360 hybrid grouper fish, each with a similar initial weight (average W_0_ = 532.28 ± 27.31 g), across twelve 500 L white cylindrical plastic culture vats. These vats were divided into four groups: one control and three probiotic-supplemented groups, with each group comprising three replicates. We maintained a consistent photoperiod in each incubator, consisting of equal 12 h intervals of light and darkness. Additionally, independent oxygen supply systems were installed in each vat to ensure sufficient oxygen levels.

The feeding experiment spanned a total of six weeks. We fed the fish twice daily, at 9:00 a.m. and 5:00 p.m., until they exhibited visual signs of satiation. Post-feeding, we regularly removed any uneaten bait and waste from the tanks and ensured thorough cleaning. To maintain a stable external environment, we replaced the seawater, ensuring it was well aerated, every four days. Throughout the feeding period and subsequent challenge test, we closely monitored and maintained water quality parameters within specific ranges: temperature at 26.5 ± 1.5 °C, dissolved oxygen at 6.50 ± 0.5 mg/L, pH at 7.6 ± 0.5, total ammonia nitrogen at 0.26 ± 0.03 mg/L, and nitrite levels at 0.04 ± 0.01 mg/L.

### 2.5. Sampling Process

For this study, we subjected the groupers to a 24 h fasting period before sampling. On the sampling day, the fish were anesthetized with 100 mg/L eugenol. We then collected whole blood from the tail vein using a 1 mL syringe. The blood samples were allowed to rest at room temperature for three hours before centrifuging at 3000 rpm for 20 min at 4 °C. This process separated the serum, which we then stored at −20 °C for subsequent analysis of liver health-related enzyme activities and antioxidant capacities. Following blood collection, we performed dissections in a sterile environment. We meticulously extracted liver samples and immediately immersed them in liquid nitrogen for preservation. These samples will be analyzed later to investigate gene expressions related to immunity and lipid metabolism. 

### 2.6. Analysis of Liver Health-Related Enzyme Activities

We measured the activities of liver health-related enzymes, specifically aspartate aminotransferase (AST) and alanine aminotransferase (ALT), utilizing specific assay kits provided by Nanjing Jiancheng Bioengineering Institute, Nanjing, China.

### 2.7. Antioxidant Capacity Analysis

The antioxidant capacities, including catalase (CAT), total antioxidant capacity (T-AOC), glutathione peroxidase (GSH-Px), and malondialdehyde (MDA) levels, were assessed using commercial assay kits sourced from Nanjing Jiancheng Bioengineering Institute, Nanjing, China.

### 2.8. Expression of Immune/Lipid Metabolism-Related Genes in Liver

We evaluated the expression of immune-related genes (*il-1β, tnfα, tlr22, myd88, p65, bcl-2, bax, caspase3, caspase9*) and lipid metabolism-related genes (*lpl, aco1, g6pd, dgat2,* fas, *atgl, pparα, cpt1, srebp-1c*) in liver tissues using RT-PCR. The process began by adding 1 mL of Trizol (Thermo Fisher Scientific, Waltham, MA, USA) to a 100 mg liver tissue sample. RNA extraction followed the protocol provided by Takara (Beijing, China). We used the NanoDrop1000 to measure RNA purity and concentration and verified RNA integrity via 10 g/L agarose gel electrophoresis. Following reverse transcription using the Prime-Script TM kit (Takara Bio-company, Dalian, China), the resultant cDNA was stored at −20 °C. RT-PCR experiments utilized SYBR Premix Ex Taq TM reagent (Takara Biocompany, Dalian, China). The specific primers used are listed in Table 2. The PCR conditions were as follows: an initial denaturation at 95 °C for 30 s, followed by 40 cycles of 95 °C for 5 s, and 60 °C for 31 s. We calculated relative gene expression levels using the 2^−ΔΔCt^ method, with β-actin as the internal control.

### 2.9. Challenge Test

The *Vibrio harveyi* strain utilized for the challenge test was isolated, sequenced, and identified in our previous research [33]. For the test, we revived the strain from −80 °C storage by culturing it in tryptic soy broth (Merck, Darmstadt, Germany) at 31 °C overnight. The culture was then centrifuged (5000× *g*, 15 min, 25 °C), and the supernatant was discarded. We washed the resultant bacterial pellets three times with sterile PBS and adjusted the bacterial suspension to 1 × 10^9^ CFU/mL, equivalent to McFarland Standard No. 0.5 at a wavelength of 600 nm. Following the six-week feeding trial, we randomly selected ten fish from each vat (thirty fish per group) for the challenge test. Each fish received an intraperitoneal injection of 0.1 mL of PBS containing 1.27 × 10^8^ CFU/mL of live *V. harveyi* cells [34]. Post-injection, we monitored and recorded the mortality rates in all groups for 14 days.

### 2.10. Data Analysis

We first processed the experimental data using Excel. Subsequently, we employed one-way ANOVA and Tukey’s test to analyze growth performance parameters, serum indicators, and gene expression data. For the challenge test, mortality data were analyzed using the Kaplan–Meier method. Differences between the control and probiotic-supplemented groups were assessed using the Mantel–Cox log-rank test. Graphs were generated using GraphPad Prism 9.5(GraphPad Software, San Diego, CA). All data are presented as mean ± SEM. In the results, columns marked with different letters (a, b, c) signify statistically significant differences among the groups. A *p*-value of less than 0.05 was considered statistically significant.

## 3. Results

### 3.1. Fish growth parameters

Compared to the control group, the three probiotic groups significantly enhanced feed intake (FI), feed efficiency ratio (FER), and feed conversion ratio (FCR) in hybrid grouper (*p* < 0.05). Notably, the 6-3-1 and HAINUP40 groups also significantly improved the final body weight (FBW) and weight gain rate (WGR) of the fish (*p* < 0.05) (Table 3).

### 3.2. Liver Function Enzyme Activity Analysis

**Figure 1:**
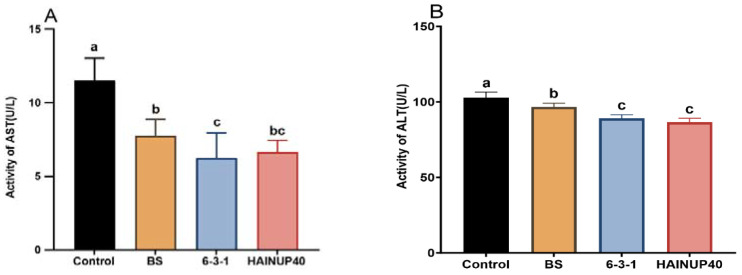
Effects of three *B. subtilis* strains on liver function enzyme activities of hybrid grouper after six-week feeding trial. (**A**) aspartate aminotransferase (AST), (**B**) alanine aminotransferase (ALT). Control: control group; BS: BS strain supplemented group; 6-3-1: 6-3-1 strain supplemented group; HAINUP40: HAINUP40 strain supplemented group. The values are presented as mean ± SEM (*n* = 9). Columns marked with different letters (a, b, c) indicate statistically significant differences between groups, as determined by ANOVA and Tukey’s test (*p* < 0.05).

As depicted in Figure 1, activities of AST (Figure 1A) and ALT (Figure 1B) were significantly lower in the BS, 6-3-1, and HAINUP40 probiotic groups compared to the control group (*p* < 0.05).

### 3.3. Antioxidant Capacity Analysis

**Figure 2:**
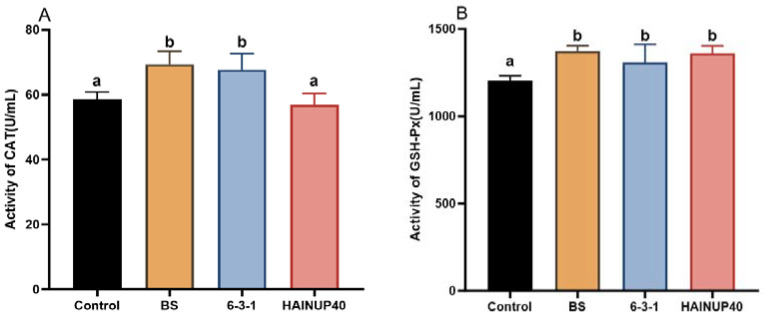

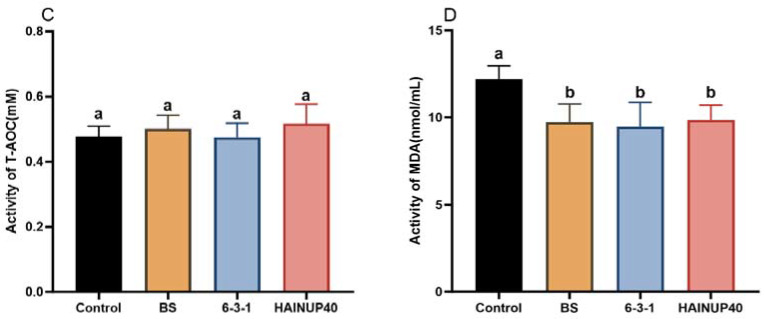
Effects of three *B. subtilis* strains on the antioxidant capacities of hybrid grouper. (**A**) catalase (CAT), (**B**) glutathione peroxidase (GSH-PX), (**C**) total antioxidant capacity (T-AOC), and (**D**) malondial dehyde (MDA). Control: control group; BS: BS strain supplemented group; 6-3-1: 6-3-1 strain supplemented group; HAINUP40: HAINUP40 strain supplemented group. The values are presented as mean ± SEM (*n* = 9). Columns marked with different letters (a, b, c) indicate statistically significant differences between groups, as determined by ANOVA and Tukey’s test (*p* < 0.05).

As shown in Figure 2, The BS group showed significant increases in CAT and GSH-PX levels compared to the control ((*p* < 0.05). Similarly, the 6-3-1 group displayed a notable increase in both CAT and GSH-PX levels (*p* < 0.05), while the HAINUP40 group demonstrated significant increases in GSH-PX levels (*p* < 0.05). All probiotic groups noted a significant reduction in MDA levels relative to the control group (*p* < 0.05).

### 3.4. Expression of Immunity-Related Genes in Liver

**Figure 3:**
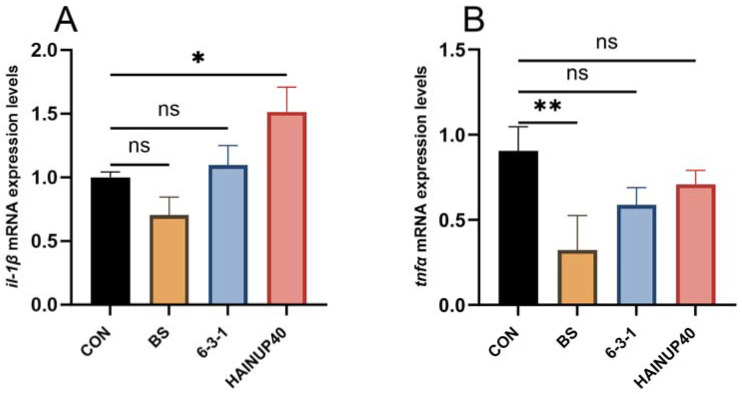

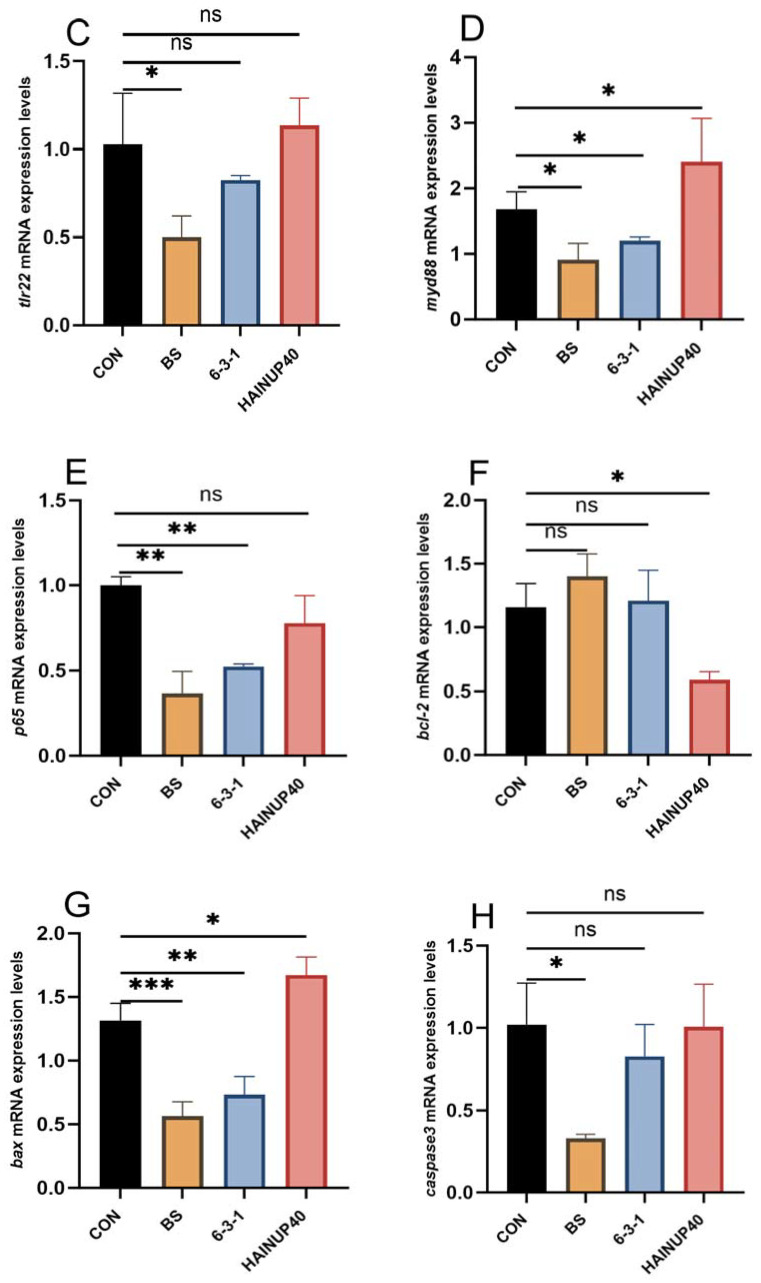

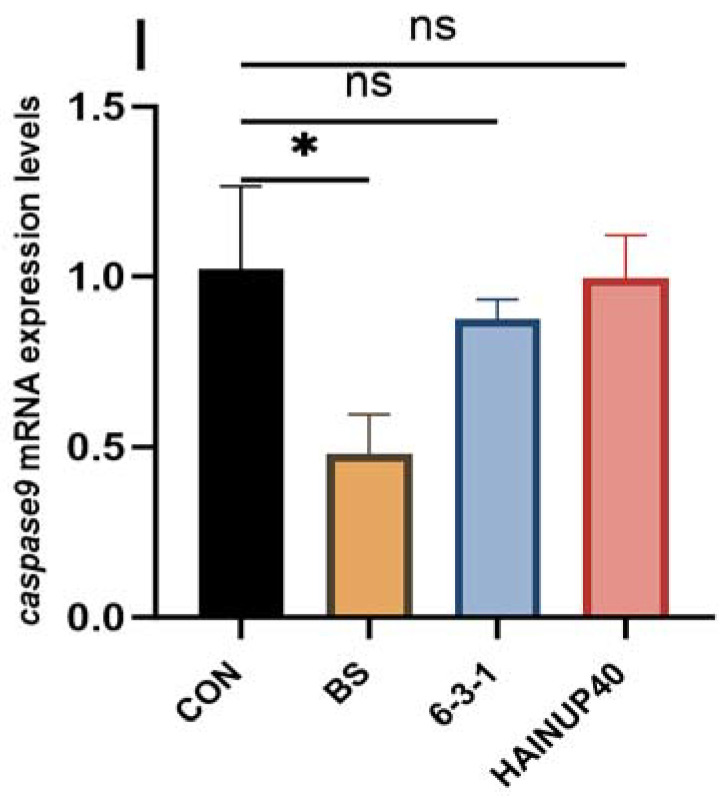
Effects of three *B. subtilis* strains on immunity-related gene expression in hybrid grouper liver. (**A**) *il-1β*: interleukin-1 beta, (**B**) *tnfα*: tumor necrosis factor alpha, (**C**) *tlr22*: toll-like receptor 22, (**D**) *myd88*: myeloid differentiation primary response 88, (**E**) *p65*: nuclear factor kappa b subunit p65, (**F**) *bcl-2*: b-cell lymphoma 2, (**G**) *bax*: bcl2 associated x, apoptosis regulator, (**H**) *caspase3*: apoptosis-related cysteine peptidase, (**I**) *caspase9*: apoptosis-related cysteine peptidase. The notation “ns” indicates no significant differences, whereas “*” marks significant differences. The values are presented as mean ± SEM (*n* = 9). Statistical significance is denoted as * *p* < 0.05, ** *p* < 0.01, and *** *p* < 0.001. The experimental groups include the control group (CON), BS strain supplemented group (BS), 6-3-1 strain supplemented group (6-3-1), and HAINUP40 strain supplemented group (HAINUP40).

As shown in Figure 3, The BS group showed a significant decrease in the expression of *tnfα*, *tlr22*, *myd88, p65, bax*, *caspase3*, and *caspase9* genes (*p* < 0.05). The 6-3-1 group exhibited a significant reduction in *myd88, p65, bax* expression (*p* < 0.05). In contrast, the HAINUP40 group displayed significant increases in *il-1β*, *myd88*, *bax* expression and a significant decrease in *bcl-2* gene expression (*p* < 0.05).

### 3.5. Expression of Lipid Metabolism-Related Genes in Liver

**Figure 4:**
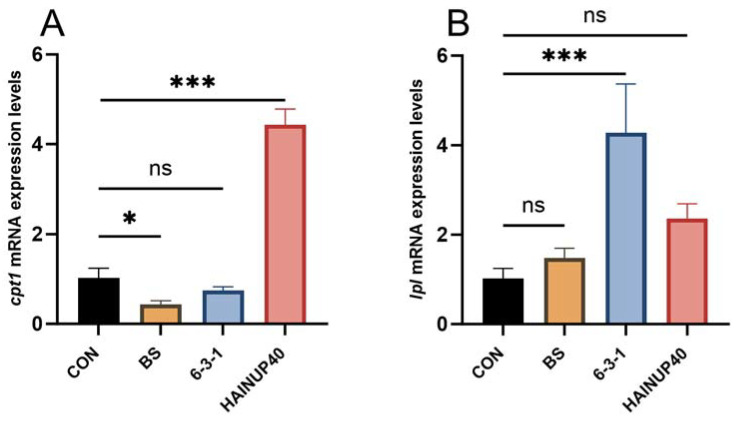

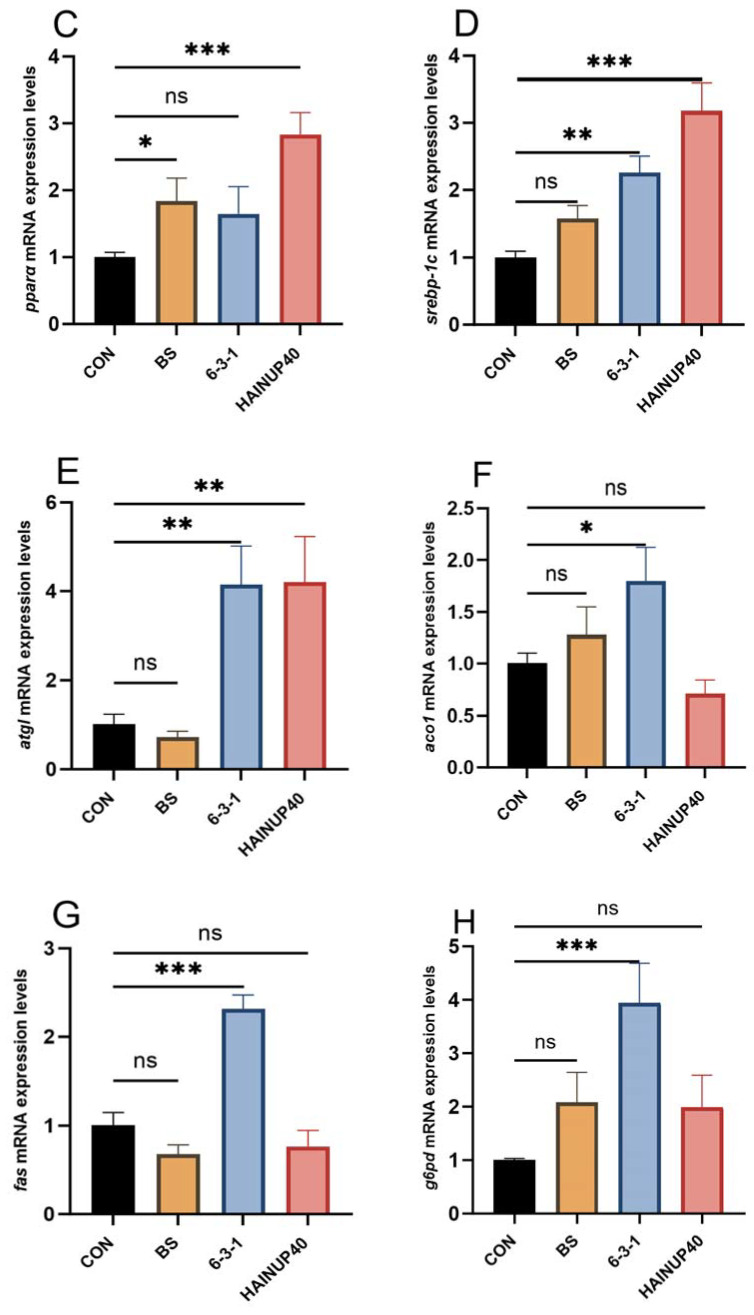

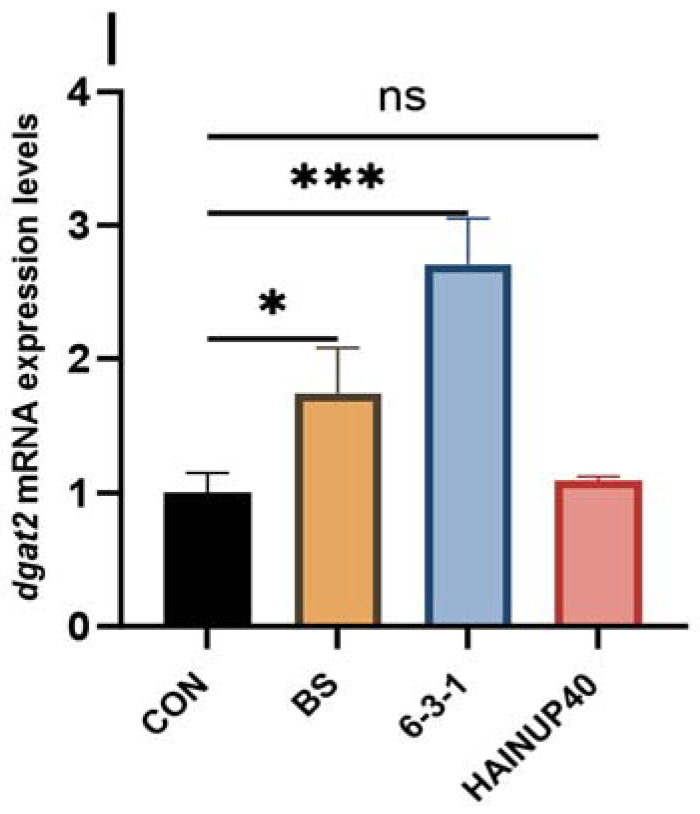
Effects of three *B. subtilis* strains on the expression of lipid metabolism-related genes in hybrid grouper liver. (**A**) *cpt1*: carnitine palmitoyltransferase1; (**B**) *lpl*: lipoprotein lipase; (**C**) *pparα*: peroxisome proliferator-activated receptor alpha; (**D**) *srebp-1c*: sterol regulatory element binding protein-1; (**E**) *atgl*: adipose triglyceride lipase; (**F**) *aco1*: acyl-CoA oxidase1; (**G**) *fas*: fatty acid synthase; (**H**) *g6pd*: glucose 6-phosphate dehydrogenase; (**I**) *dgat2*: diacylgycerol acyltransferase 2. The values are presented as mean ± SEM (*n* = 9); “ns” represents nonsignificant differences and “*” represents significant differences. * *p* < 0.05, ** *p* <0.01, *** *p* <0.001. Control: control group; BS: BS strain supplemented group; 6-3-1: 6-3-1 strain supplemented group; HAINUP40: HAINUP40 strain supplemented group.

As shown in Figure 4, in the BS group, dgat2 expression significantly increased (*p* < 0.05), while other genes either did not change significantly or were downregulated (e.g., *cpt1*, *p* < 0.05). The 6-3-1 group saw significant upregulation in seven genes (*aco1*, *atgl*, *dgat2*, *fas*, *g6pd*, *lpl*, and *srebp-1c*) (*p* < 0.05), and the HAINUP40 group showed significant upregulation in four genes (*atgl*, *cpt1*, *pparα*, and *srebp-1c*) (*p* < 0.05).

### 3.6. Challenge Test

**Figure 5:**
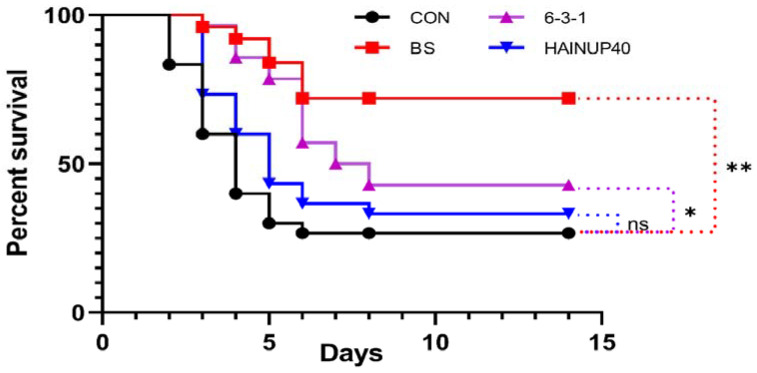
The survival rate of each group after adding different probiotic groups following the challenge experiment. After the challenge was completed, the survival status of the experimental group was monitored daily and its survival percentage calculated; “*” represents significant differences. * *p* < 0.05, ** *p* <0.01. "ns" indicates no significant difference between groups.

As shown in Figure 5, the BS and 6-3-1 groups exhibited significantly higher survival rates than the control group. Specifically, the BS group had a survival rate of 72.0%, and the 6-3-1 group had 42.9%. Conversely, the survival rate of the HAINUP40 group (33.3%) did not differ significantly from the control group. These results indicated that the BS and 6-3-1 groups had a higher likelihood of survival compared to the control group, while the HAINUP40 group showed no significant difference in survival rate.

## 4. Discussion

In recent years, the use of dietary supplements, including probiotics and medicinal plants or their derivatives, has significantly increased in aquaculture. These supplements are primarily used to enhance growth, boost immunity, and strengthen resistance against diseases and environmental stressors [35,36,37,38]. Growing concerns over human health, environmental pollution, and antibiotic resistance have spurred research into alternative growth promotion methods in fish and shrimp farming. Probiotics have gained attention as an ecofriendly and residue-free option, with several studies highlighting their effectiveness in improving the growth and health of aquatic organisms [39,40]. Various strains of *B. subtilis* have been explored as probiotics in aquaculture, but it is important to note that bacteria often display strain-specific properties. This means that different strains of the same bacterial species can have varying effects and mechanisms on the host organism [41]. Increasingly, researchers are focusing on identifying host-derived probiotics, hypothesizing that these are better adapted to the host environment and therefore more effective than nonhost-derived probiotics. However, empirical studies confirming the superiority of host-derived probiotics are scarce. This study aims to investigate and compare the effects and mechanisms of three different *B. subtilis* strains on the same species of grouper. The findings enhance understanding of the diverse impacts and mechanisms of various probiotic strains on fish.

The growth-promoting effects of *B. subtilis* strains have been have demonstrated in numerous finfishes including Nile tilapia (*O. niloticus*), tongue sole (*Cynoglossus semilaevis*), grass carp (*Ctenopharyngodon idellus*), juvenile large yellow croaker (*Larimichthys crocea*), Chinese perch (*Siniperca chuatsi*), largemouth bass (*Micropterus salmoides*), and hybrid grouper (*Epinephelus fuscoguttatus*♀ *× Epinephelus lanceolatus*♂) [42,43,44,45,46,47]. Particularly, two separate studies on hybrid groupers showed that fish fed *B. subtilis* strain GPSAK9 and strain 7K showed significantly improved fish growth in comparison to those fed the basal diet [48]. Our research aligned with these findings; all three *B. subtilis* strain supplemented groups displayed significantly enhanced feed utilization parameters (FI, FER, FCR) (*p* < 0.05). Previous research has also demonstrated that certain probiotic traits, such as growth-promoting effects, may be widely shared among most strains within a genus. Notably, in our study, the BS group did not exhibit a significant increase in FBW and WGR (*p* > 0.05) in comparison to the control group, as did the 6-3-1 and HAINUP40 groups. This observed variance in growth promotion effects among the different *B. subtilis* strains may be attributed to the strain-specific effects of probiotics. It is interesting to notice that although the BS strain showed the least growth-promoting effects among the three *B. subtilis* strains, it was also the strain with the best antidisease effects. Therefore, we propose that strains with high disease protection effects may not necessarily exhibit growth-promoting properties simultaneously [49].

Serum biochemical indices are critical for assessing the physiological status of enzyme systems in organisms and are extensively used in clinical diagnostics to evaluate fish health [50]. AST and ALT serve as biochemical markers for liver damage assessment under various physiological conditions [51]. Typically localized in liver cells, these enzymes leak into the bloodstream when liver cells are damaged, leading to elevated serum AST and ALT levels. Thus, increased levels of these enzymes are indicative of liver dysfunction in aquatic organisms [52]. In our study, we observed a significant reduction in serum AST and ALT levels in fish groups receiving *B. subtilis* probiotics compared to the control group (*p* < 0.05). Similarly, *B. subtilis* strain GPSAK9 and 1-C-7 significantly reduced the AST and ALT levels in liver of hybrid grouper and serum of Chinese perch (*Siniperca chuatsi*), respectively [43]. These results suggested a positive impact of *B. subtilis* strains on the liver health of hybrid grouper [48].

Testing antioxidant capacity is an effective method for assessing fish health [53]. Antioxidant enzymes like SOD, CAT, and GSH-Px play key roles in mitigating oxidative stress. SOD facilitates the breakdown of superoxide radicals into oxygen and hydrogen peroxide [54], while CAT decomposes hydrogen peroxide into water and oxygen [55]. GSH-Px reduces harmful peroxides to nontoxic compounds and converts hydrogen peroxide into water [56]. T-AOC reflects the collective antioxidant potential of various substances and enzymes in the body [47]. MDA is a crucial marker of lipid peroxidation in vivo, with increased levels indicating cellular damage [57]. The administration of *B. subtilis* strain GPSAK9 led to significant upregulation of serum SOD, CAT, GSH-Px, and T-AOC levels in hybrid grouper [48]. *B. subtilis* strain 7k significantly enhanced the serum SOD activity in hybrid grouper [58]. In alignment with these studies, our research found that all three *B. subtilis* strains enhanced a certain array of antioxidant capacity parameters. Specifically, improving all four tested antioxidant parameters, BS and 6-3-1 strain showed the best antioxidant effects within three strains. These findings indicated that different *B. subtilis* strains could positively influence antioxidant enzyme activities of hybrid grouper at various levels. The antioxidant effects of various *B. subtilis* stains were also reported in many other fish species [44,48,58]. However, due to the lack of overlap in the fish species studied and the antioxidant parameters measured across different studies, understanding the regulatory effects of specific strains on the antioxidant capacity of organisms remains limited.

Abnormal lipid metabolism can significantly impact liver function, leading to severe health and growth issues in fish [59,60]. Genes such as *srebp-1c*, *fas*, *g6pd*, *dgat2*, *atgl*, *cpt1*, *aco1*, *lpl*, and *pparα* play crucial roles in normal hepatic lipid metabolism in fish [61]. *Srebp-1c*, *fas*, *g6pd*, and *dgat2* are lipogenic genes involved in liver fat formation [62,63], while *atgl*, *cpt1*, and *aco-1* are associated with mitochondrial fatty acid β-oxidation [64,65]; LPL is a key enzyme in lipoprotein tag hydrolysis [66], and activation of *pparα* can suppress *fas* mRNA expression [67]. Thus, reducing *srebp*-1c, *fas*, *g6pd*, and *dgat2* levels and increasing *atgl*, *cpt-1*, *aco-1*, *lpl*, and *pparα* can mitigate lipid accumulation, enhancing fish health and growth by improving lipid metabolism [68]. For example, improved growth performance (FBW and WGR) in hybrid grouper (*E. fuscoguttatus*♀ × *E. lanceolatus*♂) has been linked with increased expression of lipolysis-related genes (*pparα*, *cpt1*, *lpl*) and decreased expression of adipogenesis-related genes (*srebp-1c*, *g6pd*, *fas*, *dgat2*) [68,69]. However, our study suggested a different relationship between fish growth and lipid metabolism-related gene expression: increased growth seems more related to the number of significantly altered genes rather than the specific upregulation or downregulation of certain gene categories. For instance, the 6-3-1 and HAINUP40 groups, which showed the largest number of significantly changed metabolism-related genes, also demonstrated the best growth performance (FBW, WGR). In the 6-3-1 group, both lipolysis-related (*aco1*, *atgl*, *lpl*) and adipogenesis-related genes (*srebp-1c*, *g6pd*, *fas*, *dgat2*) were upregulated (*p* < 0.05). Similarly, the HAINUP40 group exhibited upregulation in both lipolysis-related genes (*atgl*, *cpt-1*, *pparα*) and an adipogenesis-related gene (*srebp-1c*) (*p* < 0.05). In contrast, the BS group, which showed the fewest significant changes in metabolism-related genes, displayed weaker growth-promoting effects. In addition, a study of *B. subtilis* strain HGcc-1’s effect on high fat diet zebra fish (*Danio rerio*) revealed that although HGcc-1 supplementation significantly decreased the expression of lipogenesis genes (*c/ebpα*, *fas*, *acc1*, and *dgat2*) and increased the expression of lipolysis genes (*ucp2*, *pparα*), no significant differences were observed in growth performances of the HGcc-1 supplemented group and non-HGcc-1 supplemented group [70]. These observations not only indicated different lipid metabolism regulatory mechanisms of various *B. subtilis* strains, they also suggested that fish growth is regulated by a complex network of metabolism-related genes [71]. The expression of these genes is a dynamic process [72], and simple upregulation or downregulation cannot fully reflect the organism’s metabolic status or be directly correlated with growth performance [73].

*V. harveyi* is a prevalent and severe pathogen in grouper fish, known to cause liver injury and inflammation, which in turn can exacerbate liver damage [74,75]. Apoptosis, particularly under pathological conditions, can act as a pro-inflammatory process since the lipids from apoptotic cells may attract inflammatory cells [76]. Hence, maintaining a balance in inflammation and apoptosis is vital for liver health. In our study, we observed that the anti-inflammatory and anti-apoptotic effects exerted by probiotics seemed linked to their protective role against pathogen infections in fish. Specifically, the downregulation of pro-inflammatory and pro-apoptotic genes in the liver correlated with improved fish survival following *V. harveyi* infection. In the BS group, four pro-inflammatory genes (*tlr22*, *tnfα, myd88, p65*) and three pro-apoptotic genes (*bax*, *caspase3*, *caspase9*) were significantly downregulated compared to the control group (*p* < 0.05). Similarly, in the 6-3-1 group, the expression of one pro-inflammatory gene (*myd88, p65*) and one pro-apoptotic gene (*bax*) was markedly reduced (*p* < 0.01). Notably, both BS and 6-3-1 groups showed significant improvements in survival rates post-*V. harveyi* infection, indicating that downregulating pro-inflammatory and pro-apoptotic genes might enhance survival capabilities after infection. Conversely, the HAINUP40 group displayed upregulation in both pro-inflammatory and pro-apoptotic genes, coupled with the downregulation of anti-apoptotic genes (*p* < 0.05), yet survival rates in this group did not significantly differ from the control group. The contrast between the HAINUP40 and BS strains is intriguing: HAINUP40 showed the most substantial effects in promoting growth and upregulating lipid metabolic genes but did not offer protection against pathogen infection. In contrast, the BS strain, despite exhibiting the least growth-promoting effects and nonsignificant regulatory impact on lipid metabolic genes, demonstrated the strongest protective effects against pathogen infection and inflammation/apoptosis. This suggested that some probiotic strains might enhance host fish disease resistance without significantly promoting growth [49]. Within the three strains, host-derived 6-3-1 displayed the best overall benefits: growth promoting, disease protective, and anti-inflammatory/apoptotic. The all-around benefits of host-derived *B. subtilis* strains were also reported in [48,58], where two other *B. subtilis* strains—GPSAK9 and 7k—not only significantly improved the growth performance of hybrid grouper, but also significantly enhanced fish resistance against *V. harvey* (strain GPSAK9) and Singapore grouper iridovirus (strain 7k). But disparity also exists. GPSAK9 and 7k were reported to significantly upregulate the expression of inflammatory genes (*tgfβ* by strain GPSAK9 and *ilβ and tnfα* by strain 7k), while as 6-3-1 downregulated inflammatory genes (*myd88, p65*) [48,58]. Because the number of immune-related genes was quite limited in each study, it is difficult to tell whether the disparities in the above studies were due to the different function mechanisms of specific probiotic strains or not. Further research is needed to confirm the link between a given mechanism and the benefits.

## 5. Conclusions

This study rigorously evaluated the effects and functional mechanisms of three *B. subtilis* strains, each derived from different sources, on hybrid grouper. Over a six-week supplementation period, these strains demonstrated diverse benefits, including enhanced growth, liver health improvement, boosted antioxidant capacity, optimized lipid metabolism, and reduced inflammation and apoptosis. Notably, they also enhanced disease resistance against *V. harveyi*. Among the strains, 6-3-1, the sole host-derived probiotic, exhibited the most comprehensive benefits, significantly enhancing both growth and disease resistance. The BS strain stood out for its superior antioxidative and antidisease capabilities, while the HAINUP40 strain excelled in promoting growth. Our findings highlighted the distinct modulatory impacts that different strains of the same bacterial species can have on host fish. The superior efficacy of the host-derived 6-3-1 strain underlined the potential benefits of host-specific probiotics. However, the study also indicated the necessity for further research involving a broader range of probiotic strains and host species. Such comprehensive studies are essential to conclusively ascertain the advantages of host-derived probiotics over those from other sources. In conclusion, this investigation not only advances our understanding of probiotic applications in aquaculture but also underscores the importance of tailored probiotic strategies for enhancing fish health and productivity, thereby contributing valuable insights to the field of fish and shellfish immunology.

## Figures and Tables

**Table 1:** Ingredients and approximate composition of basic diet.

Ingredients	%
Red fishmeal	40.00
Casein	11.54
Gelatine	2.85
Wheat flour	20.00
Fish oil	4.66
Soy lecithin	2.00
Calcium monophosphate	1.00
Vitamin premix ^a^	0.22
Mineral premix ^b^	0.56
Antioxidants	0.06
Choline chloride	0.50
Vitamin C	0.09
Cellulose microcrystalline	16.52
Proximate composition (in dry matter)	
Crude protein	48.86
Crude lipid	10.75
Moisture	9.58

^a^ vitamin B_1_: 18.50 g; vitamin B_2_: 18.67 g; vitamin B_6_: 32.43 g; vitamin B_12_: 0.17 g; vitamin K: 3.43 g; vitamin E: 65.95 g; retinyl acetate: 6.77 g; vitamin D: 34.33 g; nicotinic acid: 68.33 g; D-calcium pantothenate: 39.87 g; biotin: 18.53; folic acid: 3.97 g; inositol: 101.04 g; cellulose: 598.62 g; the remaining ingredient was corn starch. ^b^ AlCl_3_·6H_2_O: 1 g; CoCl_2_·6H_2_O: 0.1 g; CaCO_3_: 350 g; CuCl_2_·2H_2_O: 1 g; FeSO_4_·7H_2_O: 2 g; KH_2_PO_4_: 200 g; MgSO_4_·7H_2_O: 10 g; MnSO_4_·7H_2_O: 2 g; NaH_2_PO_4_·H_2_O: 200 g; NaCl: 12 g; KF: 1 g; NaMoO_4_·2H_2_O: 0.5 g; NaSeO_3_: 0.4 g; KI: 0.1 g; zeolite powder: 219.9 g.

**Table 2:** Sequences of primers for qPCR.

Gene	Primer Name	Primer Sequence 5′–3′
*atgl*	*atgl*-F	ATTGAGCACCTTCCACCCA
	*atgl*-R	CCGAATCCATCCCACATCTT
*aco-1*	*aco1*-F	CGGCATGGACTTCCTGTATG
	*aco1*-R	CCTGGTGTGCGTGTTGTGTT
*fas*	*fas*-F	CGGGTGTCTACATTGGGGTG
	*fas*-R	GAATAGCGTGGAAGGCGTTT
*dgat2*	*dgat2*-F	CATCTTCTGCTTTGGTGCTTTC
	*dgat2*-R	GCATTTCCCGTCCCGTTA
*g6pd*	*g6pd*-F	GCTTCACATCCTTGTATCTGCTC
	*g6pd*-R	GCGTTCCTTTCATTCTCCG
*pparα*	*pparα*-F	CATCGACAATGACGCCCTC
	*pparα*-R	GCCGCTATCCCGTAAACAAC
*srebp-1c*	*srebp1c*-F	AGCTGTTTACACACTCCACTCG
	*srebp1c*-R	ACTGTGGAAGCTACAGGGCA
*cpt1*	*cpt1*-F	TCCTTACCGTTGGTCCCTCT
	*cpt1*-R	CTTTCCATCTGCTGCTCTATCTC
*lpl*	*lpl*-F	TTCAACAGCACCTCCAAAACC
	*lpl*-R	GTGAGCCAGTCCACCACGAT
*il-1β*	*il-1β*-F	CGACATGGTGCGGTTTC
	*il-1β*-R	TCTGTAGCGGCTGGTGG
*tnfα*	*tnfα*-F	GTGGCCTACACGACTGCACC
	*tnfα*-R	TACAAAGGGCCACAGTGAGA
*tlt22*	*tlr22*-F	CGAGCCAGGTAAACCCATCA
	*tlr22*-R	CTCATCAAACAGGCGGAAGC
*myd88*	*myd88*-F	CGAGCCAGGTAAACCCATCA
	*myd88*-R	CTCATCAAACAGGCGGAAGC
*p65*	*p65*-F	GGGTGTGTATGGATGGGG
	*p65*-R	TGGCTGGGTGGGTCTTAG
*bcl-2*	*bcl-2*-F	TTAGGTCGCAGTGAGT
	*bcl-2*-R	CATAGATGGGGAAGAG
*bax*	*bax*-F	GCCAGCAGCACATCACTTCC
	*bax*-R	AGCCACCGTAGTTCAAGCAGTT
*caspase3*	*caspase3*-F	CGCAAAGAGTAGCGACGGA
	*caspase3*-R	CGATGCTGGGGAAATTCAGAC
*caspase9*	*caspase9*-F	TTTTCCTGGTTATGTTTCGTGG
	*caspase9*-R	TTGCTTGTAGAGCCCTTTTGC
*β-actin*	*β-actin*-F	TACGAGCTGCCTGACGGACA
	*β-actin*-R	GGCTGTGATCTCCTTCTGC

**Table 3:** Effects of three *B. subtilis* strains on growth parameters of hybrid grouper after six-week feeding trial.

Parameter	Control	BS	6-3-1	HAINUP40	*p*-Value
IBW, g	540.80 ± 23.17	531.67 ± 37.51	531.33 ± 20.50	525.33 ± 28.07	0.198
FBW, g	1344.00 ± 39.40 ^a^	1323.33 ± 24.34 ^a^	1422.00 ± 14.11 ^b^	1435.33 ± 21.55 ^b^	<0.001
FI, %	755.91 ± 28.64 ^a^	592.84 ± 8.88 ^b^	547.04 ± 8.19 ^b^	553.56 ± 21.57 ^b^	<0.001
FER, %	106.23 ± 1.08 ^a^	133.54 ± 3.60 ^b^	162.83 ± 1.81 ^c^	164.53 ± 5.75 ^c^	<0.001
FCR, %	94.00 ± 1.0 ^a^	75.00 ± 2.0 ^b^	58.00 ± 1.0 ^c^	61.00 ± 2.0 ^c^	<0.001
WGR, %	148.59 ± 8.29 ^a^	148.92 ± 4.85 ^a^	167.66 ± 2.69 ^b^	173.24 ± 1.40 ^b^	<0.001

IBW: initial body weight; FBW: final body weight; FI: feed intake; FER: feed efficiency ratio; FCR: feed conversion rates; WGR: weight gain rate. The mean ± SEM is used to present the data. Values that have different superscripts within the same row exhibit significant differences (*p* < 0.05).

## Data Availability

For additional information or clarification, readers are encouraged to contact the authors directly.

## Abbreviations

IBW	initial body weight	ALT	alanine aminotransferase
FBW	final body weight	AST	aspartate aminotransferase
FI	feed intake	CAT	catalase
FER	feed efficiency rate	T-AOC	total antioxidant capacity
FCR	feed conversion rate	GSH-Px	glutathione peroxidase
WGR	weight gain rate	MDA	malondialdehyde
*ucp2*	mitochondrial uncoupling protein 2	*il-1β*	interleukin-1 beta
*acc1*	acetyl coenzyme a carboxylase 1	*bcl-2*	b-cell lymphoma 2
*atgl*	adipose triglyceride lipase	*tlr22*	toll-like receptor 22
*aco1*	acyl-CoA oxidase1	*V. harveyi*	*Vibrio harveyi*
*fas*	fatty acid synthase	*O. niloticus*	*Oreochromis niloticus*
*g6pd*	glucose 6-phosphate dehydrogenase	*B. subtilis*	*Bacillus subtilis*
*dgat2*	diacylgycerol acyltransferase 2		
*c/ebpα*	CCAAT/enhancer binding protein α		
*tgfβ*	transforming growth factor beta		
*myd88*	myeloid differentiation primary response 88		
*p65*	nuclear factor kappa b subunit p65		
*tnfα*	tumor necrosis factor alpha		
*bax*	bcl2 associated x, apoptosis regulator		
*caspase3*	apoptosis-related cysteine peptidase		
*caspase9*	apoptosis-related cysteine peptidase		
*cpt1*	carnitine palmitoyltransferase1		
*lpl*	lipoprotein lipase		
*pparα*	peroxisome proliferator-activated receptor alpha		
*srebp-1c*	sterol regulatory element binding protein-1		
